# Chinese Registry of rheumatoid arthritis (CREDIT): II. prevalence and risk factors of major comorbidities in Chinese patients with rheumatoid arthritis

**DOI:** 10.1186/s13075-017-1457-z

**Published:** 2017-11-15

**Authors:** Shangyi Jin, Mengtao Li, Yongfei Fang, Qin Li, Ju Liu, Xinwang Duan, Yi Liu, Rui Wu, Xiaofei Shi, Yongfu Wang, Zhenyu Jiang, Yanhong Wang, Chen Yu, Qian Wang, Xinping Tian, Yan Zhao, Xiaofeng Zeng, CREDIT Co-authors

**Affiliations:** 1 Department of Rheumatology, Peking Union Medical College Hospital, Peking Union Medical College and Chinese Academy of Medical Sciences, Key Laboratory of Rheumatology and Clinical Immunology, Ministry of Education, No. 1 Shuaifuyuan, Wangfujing Ave, 100730 Beijing, China; 2 Department of Rheumatology, Southwest Hospital, Third Military Medical University, Chongqing, China; 3 Department of Rheumatology, The First People’s Hospital of Yunnan Province, Kunming, Yunnan China; 4 Department of Rheumatology, Jiujiang No.1 People’s Hospital, Jiujiang, Jiangxi China; 5 Department of Rheumatology, The Second Affiliated Hospital of Nanchang University, Nanchang, Jiangxi China; 6 Department of Rheumatology, West China Hospital, Sichuan University, Chengdu, Sichuan China; 7 Department of Rheumatology, The First Affiliated Hospital of Nanchang University, Nanchang, Jiangxi China; 8 Department of Rheumatology, The First Affiliated Hospital of Henan University of Science and Technology, Luoyang, Henan China; 9 Department of Rheumatology, The First Affiliated Hospital of Baotou Medical College, Inner Mongolia University of Science and Technology, Baotou, Inner Mongolia China; 10 Department of Rheumatology, The First Hospital of Jilin University, Changchun, Jilin China; 11 Department of Epidemiology and Bio-statistics, Institute of Basic Medical Sciences, Chinese Academy of Medical Sciences, School of Basic Medicine, Peking Union Medical College, Beijing, China

**Keywords:** Rheumatoid arthritis, Comorbidity, Cardiovascular disease, Fragility fracture, Malignancy, Risk factor, Methotrexate

## Abstract

**Background:**

Rheumatoid arthritis patients are at higher risk of developing comorbidities. The main objective of this study was to evaluate the prevalence of major comorbidities in Chinese rheumatoid arthritis patients. We also aimed to identify factors associated with these comorbidities.

**Methods:**

Baseline demographic, clinical characteristics and comorbidity data from RA patients enrolled in the Chinese Registry of rhEumatoiD arthrITis (CREDIT) from Nov 2016 to August 2017 were presented and compared with those from five other registries across the world. Possible factors related to three major comorbidities (cardiovascular disease, fragility fracture and malignancy) were identified using multivariate logistic regression analyses.

**Results:**

A total of 13,210 RA patients were included (80.6% female, mean age 52.9 years and median RA duration 4.0 years). Baseline prevalence rates of major comorbidities were calculated: CVD, 2.2% (95% CI 2.0–2.5%); fragility fracture, 1.7% (95% CI 1.5–1.9%); malignancy, 0.6% (95% CI 0.5–0.7%); overall major comorbidities, 4.2% (95% CI 3.9–4.6%). Advanced age was associated with all comorbidities. Male gender and disease duration were positively related to CVD. Female sex and longer disease duration were potential risk factors for fragility fractures. Ever use of methotrexate (MTX) was negatively related to baseline comorbidities.

**Conclusions:**

Patients with rheumatoid arthritis in China have similar prevalence of comorbidities with other Asian countries. Advanced age and long disease duration are possible risk factors for comorbidities. On the contrary, MTX may protect RA patients from several major comorbidities, supporting its central role in the management of rheumatoid arthritis.

**Electronic supplementary material:**

The online version of this article (doi:10.1186/s13075-017-1457-z) contains supplementary material, which is available to authorized users.

## Background

Rheumatoid arthritis (RA) is a common systemic autoimmune disease characterized by synovial hyperplasia, chronic joint inflammation, and extra-articular manifestations. In addition to joint deformity and disability that are directly related to joint inflammation, patients with RA are also reported to have higher prevalence of comorbidities such as cardiovascular disease, osteoporotic fracture and malignancy [1–10]. The presence of comorbidities may increase the mortality of RA patients and affect their treatment strategies, resulting in worse outcomes [11–15]. According to this, the prediction and management of comorbidities have been increasingly important in the long-term management of RA [16].

To better understand the presence and development of comorbidities in RA patients, several registries and cohorts all over the world have included related information in their data collection. Baseline data for prior and current comorbidities are collected at enrollment, and during follow-up visits incident conditions are captured [17]. These data provide information about the prevalence, incidence, risk factors and other characteristics of selected comorbidities, which may further be referred by rheumatologists to improve comorbidity detection and management strategies.

In China, rheumatoid arthritis has an estimated prevalence of 0.42%, affecting more than 5 million patients by 2013 [18]. However, little is known about the comorbidities of RA in this large RA population. Previous studies are restricted to relatively small sample size and local data sources [19, 20]. The Chinese Registry of rhEumatoiD arthrITis (CREDIT) is the first nationwide multicenter prospective RA cohort in China. In this study, based on the preliminary results from CREDIT, we evaluated the nationwide prevalence of major comorbidities in Chinese RA patients, as well as the differences between patients with or without these comorbidities. By conducting this study, we hope to provide a little supplement to the relatively limited data in Asia, especially in China.

## Methods

### Study population

The Chinese Registry of rhEumatoiD arthrITis (CREDIT) established in Nov 2016 is the first nationwide, multicenter prospective registry of rheumatoid arthritis patients in China. Its goal is to provide “real-world” data regarding clinical characteristics and long-term treatment outcomes of RA in China. Consecutive patients visiting the participating centers were invited to enroll in the registry if they fulfilled the 2010 American College of Rheumatology classification criteria for RA and were able to understand and complete the questionnaires that were administered [21]. By the time of this writing in August 2017, more than 13,000 RA patients have been recruited into this cohort by rheumatologists from 173 centers (departments of rheumatology in 157 academic and 16 local hospitals), covering 31 provinces all over the country (see the map of participating centers in Additional file 1). Data are collected by rheumatologists by interviewing the patients using predefined standard online questionnaires, which include demographic data (age, gender), disease characteristics, past and present treatment for RA (types of medication, dosage and treatment course, adverse effects, etc.), as well as the presence of selected major comorbidities. Disease characteristics collected include initial fulfillment of RA diagnostic criteria, disease duration, erythrocyte sedimentation rate (ESR), C-reactive protein (CRP), seropositivity for rheumatoid factor (RF) or anti-citrullinated protein antibodies (anti-CCP), morning stiffness, tender joint count (TJC, 28 joint count), swollen joint count (SJC, 28 joint count), patient and physician global assessment (PGA and PhGA) and disease activity measured by disease activity score 28 (DAS28), SDAI, and CDAI. In the present study we aimed to analyze the occurrence of three major comorbidities in adult RA patients, so we only included patients who were aged 18 years or older and had complete data for baseline comorbidities.

Informed consent was obtained from all patients at enrollment. Ethics approval for the registry was obtained from the Medical Ethics Committee of Peking Union Medical College Hospital (PUMCH), which was accepted by all participating centers as the central institutional review board (IRB).

### Comorbidities

In the CREDIT registry, the following three comorbidities are recorded as major comorbidities of RA: cardiovascular disease (CVD), fragility fracture (osteoporotic fractures at any sites, such as vertebrae, hip and distal radius) and malignancy. Cardiovascular diseases include both coronary artery disease (CAD, consisting of angina pectoris and myocardial infarction) and stroke (ischemic or hemorrhagic). For malignancies, data on their sites and types are also recorded. Presence of these comorbidities is evaluated and recorded by rheumatologists at enrollment and each follow-up visit. Baseline comorbidity information is collected at enrollment. Rheumatologists ask their patients whether they have been diagnosed with any of the three major comorbidities by other physicians. These data are mainly based on patients’ reports, and corresponding medical records will be checked when a diagnosis of comorbidity is ambiguous.

### Statistical analysis

We analyzed the baseline characteristics of all patients included in this study using descriptive statistics (means, median, and range). Continuous variables were analyzed according to their distribution. Normally distributed continuous variables were presented as mean and standard variation, and non-normally distributed variables were presented as median and interquartile range. Categorical variables were presented as rates. Baseline overall and separate prevalence rates of the major comorbidities were calculated. In order to identify possible factors related to the presence of CVD, fracture and malignancy, we compared the patients with only one of the three major comorbidities with those without any comorbidity. Baseline variables including demographic features, clinical characteristics and medications were evaluated using univariate and multivariate logistic regression analyses. For each variable, the odds ratios (OR) and associated 95% confidence intervals (CI) were calculated.

Statistical significance was defined as *p* < 0.05. All statistical analyses were performed using SPSS software (version 23.0, IBM SPSS Inc., Armonk, NY, USA).

## Results

### Baseline characteristics and prevalence of comorbidities

A total of 13,210 patients with complete comorbidity data were included in this study (see Additional file 2). Their baseline characteristics are presented in Table 1. The mean age of these patients was 52.9 years and 80.6% of them were female. The median disease duration of RA was 4.0 years. Over 83% of the patients were seropositive for either RF or anti-CCP antibody, and the mean DAS28 was 4.5. 40.6% of the patients had been treated with glucocorticoid (GC), and approximately 55.9%, 45.9%, 30.4%, 4.4%, and 8.3% of them had received methotrexate (MTX), leflunomide (LEF), hydroxychloroquine (HCQ), sulfasalazine (SSZ) and biologic disease-modifying antirheumatic drugs (bDMARDs) therapies, respectively. The baseline characteristics of patients with or without comorbidities are also presented separately in Table 1.

**Table 1:** Baseline characteristics of study population in the CREDIT registry

	Total cohort	Without comorbidity	With comorbidity			
			Any	CVD	Fracture	Malignancy
N (%)	13,210 (100)	12,651 (95.8)	559 (4.2)	293 (2.2)	222 (1.7)	78 (0.6)
Demographic features						
Female, %	80.6	80.8	77.6	68.6	88.3	80.8
Age, y^a^	52.9 ± 13.1	52.4 ± 13.0	62.9 ± 10.8	65.6 ± 9.5	61.0 ± 11.8	60.9 ± 10.4
Age ≥ 60, %	32.7	31.2	66.9	75.4	61.7	59.0
Clinical characteristics						
RA duration, y^b^	4.0 (1.3–10.0)	4.0 (1.2–10.0)	6.3 (2.0–13.1)	6.0 (1.4–12.9)	7.7 (3.0–15.7)	4.0 (1.2–10.7)
RF/CCP+, %	83.6	83.4	86.9	86.0	86.5	89.7
ESR, mm/h^b^	34.0 (17.0–60.0)	33.0 (17.0–60.0)	41.0 (21.0–66.0)	40.0 (20.5–67.5)	39.5 (20.8–66.0)	45.0 (22.8–65.0)
CRP, mg/L^b^	10.3(3.2–30.0)	10.2 (3.2–30.0)	11.5 (3.2–35.8)	12.0 (3.3–39.2)	9.9 (3.2–35.5)	7.7 (2.8–29.7)
DAS28-CRP^a^	4.5 ± 1.7	4.4 ± 1.7	4.7 ± 1.7	4.7 ± 1.7	4.7 ± 1.8	4.6 ± 1.8
Medications						
GC ever, %	40.6	40.5	43.1	46.1	41.9	34.6
MTX ever, %	55.9	56.4	44.7	42.3	47.7	37.2
bDMARDs, %	8.3	8.2	9.3	8.9	14.4	1.3

*CVD* cardiovascular disease, *RA* rheumatoid arthritis, *RF* rheumatoid factor, *CCP* anti-citrullinated protein antibody, *DAS28* disease activity score 28, *ESR* erythrocyte sedimentation rate, *CRP* C-reactive protein, *GC* glucocorticoid, *MTX* methotrexate, *bDMARDs* biological disease-modifying antirheumatic drugs

^a^Data are presented as mean ± SD (standard deviation)

^b^Data are presented as median (IQR, interquartile range)

Table 2 shows the detailed prevalence of major comorbidities at baseline. In all, 4.2% of RA patients reported that they had been diagnosed with at least one of the three comorbidities. CVD was present in 293 (2.2%) patients in total, remarkably more prevalent in male patients (3.6% vs. 1.9%). Among all patients with CVD, 204 reported to have CAD and 108 had a history of stroke. Prior fragility fractures were reported by 222 (1.7%) at enrollment, and the prevalence rate was higher for female patients (1.9% vs. 1.0). Malignancy was the least common comorbidity among RA patients (n = 78, 0.6%) in this study, with no difference between genders. The most frequently involved organs were breast (n = 17), lung (n = 10), thyroid (n = 7), colorectum (n = 5) and stomach (n = 4) (see Additional file 3). Compared with younger subjects, patients over 60 years old had higher prevalence for all comorbidities. Overlaps between comorbidities were not prevalent in the study population, and the most common overlap was between CVD and fragility fracture (28 patients).

**Table 2:** Detailed prevalence of major comorbidities at baseline

	Total cohort (n = 13,210)		Men (n = 2558)	Women (n = 10,652)	Age < 60 y (n = 8891)	Age ≥ 60 y (n = 4319)
Comorbidities	N	Prevalence (95% CI)	N (%)	N (%)	N (%)	N (%)
Any major comorbidity	559	4.2 (3.9–4.6)	125 (4.9)	434 (4.1)	185 (2.1)	374 (8.7)
Cardiovascular disease	293	2.2 (2.0–2.5)	92 (3.6)	201 (1.9)	72 (0.8)	221 (5.1)
Stroke	108	0.8 (0.7–1.0)	40 (1.6)	68 (0.6)	23 (0.3)	85 (2.0)
Coronary artery disease^a^	204	1.5 (1.3–1.8)	59 (2.3)	145 (1.4)	50 (0.6)	154 (3.6)
Angina pectoris	132	1.0 (0.8–1.2)	34 (1.3)	98 (0.9)	38 (0.4)	94 (2.2)
Myocardial infarction	50	0.4 (0.3–0.5)	17 (0.7)	33 (0.3)	4 (0.04)	46 (1.1)
Fragility fracture	222	1.7 (1.5–1.9)	26 (1.0)	196 (1.8)	85 (1.0)	137 (3.2)
Malignancy^b^	78	0.6 (0.5–0.7)	15 (0.6)	63 (0.6)	32 (0.4)	46 (1.1)
Overlap of comorbidities						
CVD + fracture	28	0.21 (0.13–0.29)	7 (0.05)	21 (0.16)	4 (0.03)	24 (0.18)
CVD + malignancy	5	0.04 (0–0.07)	1 (0.01)	4 (0.03)	0 (0)	5 (0.04)
Fracture + malignancy	1	0.01 (0–0.05)	0 (0)	1 (0.01)	0 (0)	1 (0.01)
CVD + fracture + malignancy	0	0 (0)	0 (0)	0 (0)	0 (0)	0 (0)

*CVD* cardiovascular disease

^a^Data on detailed type of CAD were missing from 22 patients

^b^Data on organ of malignancy were missing from three RA patients with malignancies

### Factors associated with the presence of major comorbidities

As shown in Table 3, the multivariate analyses of cardiovascular disease revealed that female gender (OR 0.70, 95% CI 0.53–0.92) ever use of MTX (OR 0.77, 95% CI 0.60–1.00) were negatively related to the presence of CVD. On the contrary, patients of advanced age (OR 1.09, 95% CI 1.07–1.10) and disease duration longer than 5 years (OR 1.33, 95% CI 1.03–1.72) were more likely to have these comorbid conditions. Based on the multivariate analysis, female sex (OR 2.58, 95% CI 1.59–4.18), advanced age (OR 1.05, 95% CI 1.04–1.06), RA duration (OR 1.95, 95% CI 1.44–2.63) and bDMARDs (OR 1.79, 95% CI 1.16–2.76) were associated with history of fragility fractures. The association between MTX and decreased risk of fracture was only significant in the univariate analysis (not shown). As for malignancies, fewer factors were identified in our analyses. Only advanced age (OR 1.05, 95% CI 1.03–1.07) and MTX treatment (OR 0.57, 95% CI 0.35–-0.91) were significant associated factors in the multivariate analysis.

**Table 3:** Factors associated with major comorbidities in RA patients in multivariate logistic regression analyses

	CVD		Fragility fracture		Malignancy	
	OR (95% CI)	*P*	OR (95% CI)	*P*	OR (95% CI)	*P*
Female	0.70 (0.53–0.92)	0.01^*^	2.58 (1.59–4.18)	<0.01^*^	1.26 (0.69–2.28)	0.45
Age, y	1.09 (1.07–1.10)	<0.01^*^	1.05 (1.04–1.06)	<0.01^*^	1.05 (1.03–1.07)	<0.01^*^
RA duration						
<5 years	1 (Ref)	NA	1 (Ref)	NA	1 (Ref)	NA
≥5 years	1.33 (1.03–1.72)	0.03^*^	1.95 (1.44–2.63)	<0.01^*^	0.97 (0.61–1.56)	0.91
RF/CCP+	1.30 (0.90–.88)	0.17	1.31 (0.86–2.00)	0.22	1.95 (0.89–4.27)	0.10
DAS28	0.97 (0.90–1.05)	0.48	1.04 (0.95–1.13)	0.44	1.03 (0.89–1.19)	0.70
GC ever	1.11 (0.86–1.44)	0.42	1.05 (0.78–1.40)	0.75	0.61 (0.37–1.00)	0.05
MTX ever	0.77 (0.60–1.00)	0.05^*^	0.96 (0.72–1.28)	0.78	0.57 (0.35–0.91)	0.02^*^
bDMARDs	1.04 (0.64–1.69)	0.86	1.79 (1.16–2.76)	<0.01^*^	0.15 (0.02–1.07)	0.06

*CVD* cardiovascular disease, *OR* odds ratio, *CI* confidence interval, *RA* rheumatoid arthritis, *NA* not applicable, *RF* rheumatoid factor, *CCP* anti-citrullinated protein antibody, *DAS28* disease activity score 28, *GC* glucocorticoid, *MTX* methotrexate

^*^ *P* < 0.05

## Discussion

Patients with rheumatoid arthritis tend to have a higher risk for a number of comorbidities [2–10]. The presence of these comorbid conditions was reported to harm their long-term prognosis, and even result in shortening of life expectancy [11–14]. A population-based cohort study by Gabriel et al. reported that comorbidities increased risk of death in RA patients, with hazard ratios (HR) of 1.6 (95% CI 1.2–2.1) for cardiovascular disease and 1.9 (95% CI 1.4–2.6) for malignancy [22]. Another cohort study showed the association of hip fracture and higher mortality in RA patients (1-year mortality rates18.47% vs. 6.16%) [23]. According to these findings, how to prevent, detect and manage comorbidities properly has become a vital issue in the long-term management of RA patients. In China, CREDIT is the first RA registry to provide nationwide, multicenter data for comorbidities as well as related clinical characteristics.

In this study, we assessed the prevalence of three major comorbidities in Chinese RA patients. Among cardiovascular disease, fragility fracture, and malignancy, CVD and fragility fracture were relatively prevalent. To find the differences between RA patients and normal population, we compared our results with national governmental epidemiologic data [24]. The prevalence rates of CAD, stroke, and malignancy in general adult population were 1.02%, 1.23%, and 0.29%, compared with 1.5%, 0.8%, and 0.6% in our study. It suggests that CAD and malignancy are more common, and stroke is less common in RA patients. However, our study population was composed of more women and older patients than the general population. Due to the lack of age, gender and region-specific data, we were unable to calculate the standardized rates of comorbidities for further comparison. Future studies comparing RA patients in CREDIT and general population from community/population-based cohorts will help us to get a better understanding of this problem.

In comparison with six other large registries across the world (Table 4) [14, 17, 25–37], patients in CREDIT were younger and had shorter disease duration at baseline than those in other registries, except for ERAS and ERAN, which are two inception cohorts for patients with early RA [29]. Although baseline distributions of demographic characteristics were broadly comparable across all registries, high inter-country variability was observed in the prevalence of comorbidities. Asian RA patients in IORRA and CREDIT presented lower prevalence rates of comorbidities compared to those from USA and European cohorts, which may demonstrate the effect of geographic and ethnical factors. More studies are needed to investigate potential reasons for these observations. As for history of prior fractures, the prevalence in CREDIT and ERAS was remarkably lower than those in CORRONA, IORRA and KORONA, which might be attributed to the focus on fragility fractures in CREDIT and ERAS as well as a low diagnosis rate of subclinical fractures. Other possible sources for the variability in prevalence included disease duration, disease activity, detection of comorbidities (from self-reports or medical databases), and treatment strategies in different countries [38]. Previous studies have indicated the difficulty to do comparative work across registries [17, 37]. The differences between Chinese patients and those from other countries also suggest that it is necessary to develop special RA managing strategies in China.

**Table 4:** Baseline prevalence of comorbidities in CREDIT and other large registries

Registry	CORRONA [17, 25–27]	NOAR [17, 28]	ERAS/ERAN [14, 29]	SRR [17, 30, 31]	IORRA [17, 32, 33]	KORONA [34, 35]	CREDIT
Country	USA	UK	UK	Sweden	Japan	Korea	China
Number of patients	24,989	1564	1465/1236	18,527	11,907	4721	13,210
Female, %	76.0	69.9	66.4/67.9	70.3	82.2	85.2	80.6
Age, y	58.9 ± 13.4	55.9 ± 14.6	55.3 ± 14.6/57.0 ± 14.2	58.0^b^	55.7 ± 13.5	54.3 ± 12.2	52.9 ± 13.1
Age ≥ 60, %	47.3	50.9	NR	54.1	41.0	NR	32.7
Disease duration, y	10.1 ± 9.8	6.5 (median)	0.5 (median)	NR	8.1 ± 8.6	8.3 ± 7.6	6.8 ± 7.6
CAD, %	6.4	2.6	4.5 (ERAS)	7.9	1.5	NR^c^	1.5
Stroke, %	2.1	2.7	1.5	3.0	0.3	NR^c^	0.8
Prior fracture, %^a^	21.6^d^	NR	0.8 (ERAS)	NR	23.7	16.7^e^	1.7
Malignancy, %	6.8	8.3	3.0	12.4	1.1	NR	0.6

*NR* not reported, *CAD* coronary artery disease, *CORRONA* Consortium of Rheumatology Researchers of North America Registry, *ERAS* Early Rheumatoid Arthritis Study, *ERAN* Early Rheumatoid Arthritis Network, *SRR* Swedish Rheumatology Quality of Care Register, *NOAR* Norfolk Arthritis Register, *IORRA* Institute of Rheumatology Rheumatoid Arthritis Cohort, *KORONA* KORean Observational study Network for Arthritis, CREDIT Chinese Registry of rheumatoid arthritis

^a^Only fragility fractures were collected in CREDIT, and all types of prior fractures in other cohorts ^b^Data from 12,656 patients, and standard deviation was not reported

^c^The prevalence of cardiovascular disease was 4.0%, but no data on CAD or stroke separately

^d^Data derived from 6143 RA patients with ≥1 year follow-up

^e^Data from 3557 patients with ≥1 year follow-up

Several possible associated factors were identified by comparing baseline data between RA patients with and without the major comorbidities. Advanced age was associated with all comorbidities, consistent with the consensus that aging is a traditional risk factor for CVD, osteoporotic fracture, and malignancy. Male gender, a typical risk factor for cardiovascular diseases, was associated with the presence of CVD. Previous studies have suggested that females have higher risk of osteoporosis and osteoporotic fractures especially after menopause [39], and it was also confirmed by our finding. According to our analyses, duration of RA is the most important clinical factor related to the presence of comorbidities. As indicated by several studies, chronic inflammation resulted from long-standing autoimmune diseases might be the major reason for the increased risk of comorbidities in these diseases [11, 40]. As for medications, GC is widely believed to cause higher risk of osteoporosis and fracture [41], however, it was not related to any comorbidity in our study. We assumed that it was because we only assessed ever use of GC without considering the effect of dose, duration, cumulative exposure, and the sequence between GC use and the onset of comorbidities. In future follow-up studies, we will take these factors into consideration and investigate the relationship between GC and the risk of incidence comorbidities. In China, use of bDMARDs is generally in accordance with international guidelines [42]. However, since these drugs are not covered by the national health insurance, their high cost is also an important concern in the treatment decision-making process. In clinical practice, bDMARDs are given to patients with moderate to high disease activity, severe joint damage, and poor responses to conventional medications. Therefore, the association between bDMARDs and fractures in our results may only represent an impact of disease severity on the risk of fractures.

In our study, MTX was indicated to be a potential protective factor for comorbidities in RA patients. As suggested in previous studies [43, 44], treatment with MTX was associated with a reduced risk of cardiovascular events [relative risk (RR) 0.72, 95% CI 0.57–0.91] and related deaths (HR 0.3, 95% CI 0.2–0.7). Possible mechanisms may include an improvement in the mobility of patients as well as a decrease in their systemic inflammation. The effect of MTX on fragility fractures is still controversial. Though some studies have indicated that MTX has a positive impact on bone metabolism and bone mineral density (BMD) stabilization in RA patients, additional studies are required to determine whether this effect is sufficient to reduce their risk of fractures [41, 45]. As for the risk of malignancies, existing evidence is insufficient to make a full assessment [46]. Due to its efficacy, safety, low costs and the possibility to individualized dose and method of administration, MTX continues to be the anchor drug for RA patients even after the development of numerous bDMARDs [47]. Our study suggests that MTX might also benefit RA patients by reducing the risk of several life-threatening comorbidities, supporting its central role in the management of RA.

Our study has several limitations. First, in CREDIT, data on three major comorbidities were collected, since they were reported to have vital influence on prognosis and mortality. However, comorbidities missed in this study, such as infection and interstitial lung diseases, may also affect long-term outcomes to some extent [11]. Second, the information on comorbidities and medications in CREDIT was collected by interviewing patients and mainly based on their self-reports. Though the three selected comorbidities are well known and understandable to patients, and we attempted to minimize potential bias by further verifying the ambiguous diagnoses of comorbidities, there still might be a certain degree of inaccuracy. Third, since all data in this cross-sectional study were collected at baseline, we were unable to determine possible cause-effect relationships between rheumatoid arthritis, comorbidities, and potential risk factors.

## Conclusions

In summary, CREDIT is the first nationwide, multicenter, prospective registry of rheumatoid arthritis in China. This study presents the preliminary baseline data in terms of major comorbidities from 13,210 enrolled patients, and for the first time evaluates the prevalence of three vital comorbidities in a large, nationwide sample of Chinese RA patients. Based on our findings, RA patients with advanced age, longer disease duration, and traditional factors should be more carefully monitored for comorbidities. Methotrexate, as the anchor drug in the treatment of rheumatoid arthritis, may also protect patients against several comorbidities. On the basis of this preliminary cross-sectional study, future follow-up studies are needed for further investigating the characteristics of Chinese RA patients, as well as the incidence and predictors of major comorbidities.

## Additional files

Additional file 1: Figure S1. Map of the CREDIT registry participating centers. (DOC 3195 kb)

Additional file 2: Figure S2. Flow chart of patient selection in the present study. (DOC 51 kb)

Additional file 3: Table S1. Prevalence of malignancies in CREDIT at baseline. (DOC 39 kb)

## Abbreviations

bDMARDs: biologic disease-modifying antirheumatic drugs
CAD: coronary artery disease
CCP: Anti-citrullinated protein antibody
CI: confidence interval
CORRONA: Consortium of Rheumatology Researchers of North America Registry
CRP: C-reactive protein
CVD: cardiovascular disease
DAS28: disease activity score 28
ERAN: Early Rheumatoid Arthritis Network
ERAS: Early Rheumatoid Arthritis Study
ESR: erythrocyte sedimentation rate
GC: glucocorticoid
IORRA: Institute of Rheumatology Rheumatoid Arthritis Cohort
KORONA: KORean Observational study Network for Arthritis
MTX: methotrexate
NOAR: Norfolk Arthritis Register
NR: not reported
OR: odds ratio
RA: rheumatoid arthritis
RF: rheumatoid factor
RR: relative risk
SRR: Swedish Rheumatology Quality of Care Register
