# Supplementary material for: Chinese Registry of rheumatoid arthritis (CREDIT): II. prevalence and risk factors of major comorbidities in Chinese patients with rheumatoid arthritis
Source: Arthritis Res Ther. 2017 Nov 15;19:251. doi: 10.1186/s13075-017-1457-z (PMC5688621; doi:10.1186/s13075-017-1457-z)
Supplement: Supplementary file 1 — Map of the CREDIT registry participating centers. (DOC 3195 kb) [file 13075_2017_1457_MOESM1_ESM.doc]

**Additional file 1: Figure S1.** Map of the CREDIT registry participating centers
