# Supplementary material for: Chinese Registry of rheumatoid arthritis (CREDIT): II. prevalence and risk factors of major comorbidities in Chinese patients with rheumatoid arthritis
Source: Arthritis Res Ther. 2017 Nov 15;19:251. doi: 10.1186/s13075-017-1457-z (PMC5688621; doi:10.1186/s13075-017-1457-z)
Supplement: Supplementary file 2 — Flow chart of patient selection in the present study. (DOC 51 kb) [file 13075_2017_1457_MOESM2_ESM.doc]

**Additional file 2: Figure S2.** Flow chart of patient selection in the present study

RA patients evaluated for comorbidities (n=13210)

Patients with CVD

(n=293)

Patients under 18 years old or had no baseline comorbidity data (n=71)

RA patients enrolled in CREDIT (n=13281)

Patients with fragility fracture (n=222)

Patients with malignancy (n=78)

Patients without major comorbidity (n=12651)

Patients with only fracture (n=193)

Patients with only malignancy (n=72)

Patients with only CVD (n=260)
