# Supplementary material for: Chinese Registry of rheumatoid arthritis (CREDIT): II. prevalence and risk factors of major comorbidities in Chinese patients with rheumatoid arthritis
Source: Arthritis Res Ther. 2017 Nov 15;19:251. doi: 10.1186/s13075-017-1457-z (PMC5688621; doi:10.1186/s13075-017-1457-z)
Supplement: Supplementary file 3 — Prevalence of malignancies in CREDIT at baseline. (DOC 39 kb) [file 13075_2017_1457_MOESM3_ESM.doc]

**Additional file 3: Table S1.** Prevalence of malignancies in CREDIT at baseline

|  | **Total cohort (n=13210)** | | **Men (n=2558)** | **Women (n=10652)** |
| --- | --- | --- | --- | --- |
| **Malignancy*** | N | Prevalence (95%CI) | N(%) | N(%) |
| Breast | 17 | 0.13 (0.07-0.19) | - | 17 (0.16) |
| Lung | 10 | 0.08 (0.03-0.12) | 4 (0.16) | 6 (0.06) |
| Thyroid | 7 | 0.05 (0.01-0.09) | - | 7 (0.07) |
| Colorectum | 5 | 0.04 (0.00-0.07) | 3 (0.12) | 2 (0.02) |
| Stomach | 4 | 0.03 (0.00-0.06) | 2 (0.08) | 2 (0.02) |
| Ovary | 2 | 0.02 (0.00-0.05) | - | 2 (0.02) |
| Lymphoma | 2 | 0.02 (0.00-0.05) | 1 (0.04) | 1 (0.01) |
| Liver | 1 | 0.01 (0.00-0.05) | - | 1 (0.01) |
| Esophagus | 1 | 0.01 (0.00-0.05) | 1 (0.04) | - |
| Cervix | 1 | 0.01 (0.00-0.05) | - | 1 (0.01) |
| Others | 31 | 0.23 (0.15-0.32) | 3 (0.12) | 28 (0.3) |

* Data on organ of malignancy were missing from 3 patients.
